# Influence of oestradiol on exercise induced muscular damage and heart rate variability in a non‐trained healthy population

**DOI:** 10.1113/EP093495

**Published:** 2026-05-01

**Authors:** David Ramiro‐Cortijo, Ricardo Alonso de Celada, Pilar Rodríguez‐Rodríguez, Stefanny Lozano Gutiérrez, Leonardo Betancourt, Santiago Ruvira, Silvia M. Arribas

**Affiliations:** ^1^ Department of Physiology, Faculty of Medicine Universidad Autónoma de Madrid Madrid Spain; ^2^ Food, Oxidative Stress and Cardiovascular Health (FOSCH) Research Group Universidad Autónoma de Madrid Madrid Spain; ^3^ PhD candidate in Pharmacology and Physiology, Faculty of Medicine Universidad Autónoma de Madrid Madrid Spain; ^4^ Department of Human Body Movement Universidad Nacional de Colombia Bogotá Colombia

**Keywords:** EIMD, HRV, non‐trained, oestradiol, pain, systemic inflammation

## Abstract

This study evaluates in non‐trained healthy women the influence of oestradiol (E2) on exercise induced muscular damage (EIMD) and performance. Thirty‐six young healthy women performed a step‐exercise until exhaustion, assessing the number of repetitions, pain perception and well‐being (questionnaires, 15 min and at 48 h post‐exercise), blood pressure (sphygmomanometer) and heart rate variability (HRV; H10‐polar band) were measured at rest and 15 min post‐exercise. Plasma samples were taken at rest and 2 h post‐exercise, assessing interleukin (IL)‐1α, IL‐6, IL‐1ra, IL‐10 (multiplex ELISA), creatine kinase (CK) and lactate dehydrogenase (LDH) activities, and E2 (spectrophotometry). Statistical analysis was performed comparing women with low E2 (<40 pg/mL; *n* = 18) and high E2 (≥40 pg/mL; *n* = 18). The number of repetitions was not different between groups and did not correlate with E2. Compared to the low‐E2 group, the high‐E2 group had significantly smaller lower‐body pain perception at 2 h and scored higher in well‐being at 48 h. IL‐6 and IL‐10 levels 2 h post‐exercise did not differ between groups, but both cytokines positively correlated with E2. LDH, but not CK, was lower in the high‐E2 group and negatively correlated with E2. Blood pressure at rest negatively correlated with E2. Total pain at 48 h positively correlated with LDH and negatively with IL‐10, and the opposite correlations were found with well‐being. No differences between groups or E2 correlations were found in time‐domain or frequency‐domain HRV parameters at rest or after exercise. In conclusion, in non‐trained women, oestrogen levels influence EIMD, reducing pain perception. These effects could be related to E2 actions on membrane protection and accelerating muscle fibre regeneration through IL‐6/IL‐10 myokine signalling. According to these data, oestrogen does not appear to influence HRV, and the role of progesterone deserves further attention.

## Abstract (highlights)

- **What is the central question of this study?**
  In untrained women, does oestradiol influence exercise‐induced muscular damage (EIMD), performance or heart rate variability (HRV)?
- **What is the main finding and its importance?**
  In untrained women, oestrogen levels influence EIMD, reducing pain perception. These effects may be related to skeletal muscle membrane protection and enhanced IL‐6/IL‐10 myokine signalling. Oestrogen levels do not seem to influence HRV, either at rest or after exercise performance. The present study contributes to advancing knowledge on the influence of female sex hormones in physical exercise.

## 1 INTRODUCTION

Physical activity, especially when performed at high intensity, produces muscular damage, known as exercise‐induced muscular damage (EIMD) (Ertel et al., 2020). This process is characterized by fibre disruption and inflammation, pain and reduced strength, limiting subsequent exercise performance, and typically subsides within 24–72 h (Docherty et al., 2022). Immediately after fibre damage, the release of intracellular enzymes, such as creatine kinase (CK) and lactate dehydrogenase (LDH), takes place, and a local inflammatory response is initiated. In this process, interleukin (IL)‐6 released from skeletal muscle is one of the key players, contributing to immune cell recruitment for muscle tissue repair (Peake et al., 2015). IL‐6 also regulates the production of anti‐inflammatory cytokines, including IL‐1 receptor antagonist (IL‐1ra) and IL‐10 (Petersen & Pedersen, 2005), thereby contributing to the resolution of inflammation and muscle recovery (Pedersen, 2011; Pedersen & Fischer, 2007).

To ensure equity in research, it is essential to include female participants in studies and to account for the influence of ovarian cycle and hormonal variations on physiological responses (Cabre et al., 2024; Oosthuyse & Bosch, 2017; Oosthuyse et al., 2023). One of the relevant unsolved questions is the influence of ovarian cycle hormones on EIMD (Romero‐Parra et al., 2021). There is evidence that pro‐inflammatory cytokines tend to be higher while anti‐inflammatory ones are downregulated during the menstrual cycle phases with low oestrogen levels (Notbohm et al., 2024), and a metanalysis described a higher inflammatory response in the luteal phase (Notbohm et al., 2023). Nociceptive sensitivity in response to exercise and the release of muscle damage biomarkers, such as CK, are also sex dependent. Compared to men, pain perception lasts longer while the CK response recovers more quickly in women, both being influenced by ovarian hormones (Oosthuyse & Bosch, 2017). Performance and effort perception after exercise also seem to be influenced by the ovarian cycle. In female athletes, there is evidence of larger muscle strength during cycle phases when oestrogen levels peak (Kissow et al., 2022), although a recent meta‐analysis reported that strength appears to be marginally altered by sex hormone fluctuations along the menstrual cycle (Blagrove et al., 2020). Higher performance and wellness have been described in the middle of the ovarian cycle compared to the end (Antero et al., 2023), and lower work effort has been reported during the first half of the ovarian cycle (De Larochelambert et al., 2024). On the other hand, a meta‐analysis showed that oral contraception effects seem to be trivial in terms of performance (Elliott‐Sale et al., 2020). Overall, there are no clear conclusions on the impact of the ovarian cycle on exercise performance and EIMDs, particularly in non‐trained individuals. Possible reasons include low sample size, number and relevance of biomarkers analysed, cycle phase definition and heterogeneity of the population in terms of training level and muscle adaptation, underscoring the need for additional rigorous and comprehensive studies (Cabre et al., 2024; Colenso‐Semple et al., 2025; Oosthuyse et al., 2023; Sims et al., 2023). Our study is focused on non‐trained women and on the role of 17β‐oestradiol (E2), since it is a key regulator of metabolism, skeletal muscle growth and membrane protection activities (Oosthuyse et al., 2023).

Heart rate variability (HRV) is a valuable physiological marker to assess sympathetic (SNS) and parasympathetic (PNS) nervous system regulation. In athletes, HRV has been used to evaluate the capacity of activation and recovery (Güngör et al., 2024), training adaptation (Kiss et al., 2016), and overtraining and performance (Lundstrom et al., 2023). Existing data regarding the influence of the ovarian cycle on HRV are unconclusive. Under resting conditions, some studies suggest a larger HRV and PNS predominance in low oestrogen ovarian cycle phases, decreasing during the luteal phase (Tenan et al., 2014), others shows PNS activity predominance in the high oestrogen proliferative phase (Brar et al., 2015), and some do not evidence differences in young females, although a decrease in HRV was observed in postmenopausal women (Ramesh et al., 2022). In female athletes, a higher HRV is observed when oestrogen levels are low (Sims et al., 2021), but data in non‐trained women are lacking.

Based on the above‐mentioned gap, the aim of the present study was to evaluate in non‐trained healthy women who perform physical exercise for leisure the influence of E2 levels on different manifestations of EIMD, namely, perceived pain, inflammation and muscular damage. We also evaluated the possible influence on performance and HRV.

## 2 METHODS

### 2.1 Ethical approval

Women were given oral and written information about the procedures of participating in the study, and those interested provided a written informed consent. The study has the approval of the Research Ethics Committee of Universidad Autónoma of Madrid (Ref. CEI‐136‐2901, approved on 9 February, 2024), adhering to the ethical standards in the *Declaration of Helsinki*. Personal data are subject to the Spanish Organic Law 3/2018 of 5 December 2018, and the EU Regulation 2015/2283, which safeguards the security of the participant.

### 2.2 Participants

The participants were recruited by a non‐probabilistic strategy among female students from the degrees of Medicine, Nursing and Biomedical Engineering from Universidad Autónoma de Madrid (Spain). The inclusion criteria were healthy adults, sufficient knowledge of the Spanish language, and ≥18 years old (the legal age according to current Spanish legislation). The exclusion criteria were active smokers, body composition outside the normal range for age, polycystic ovary syndrome, chronic inflammatory conditions (diabetes, hypertension, obesity or coeliac disease), and training of total activities (>60 METs kcal/kg week). Based on these criteria, 36 eumenorrheic healthy females (Spanish origin and a median 20.0 [19.0, 23.0] years old) were recruited. All participants were non‐smokers and normotensive (<120 mmHg systolic blood pressure [SBP] and <80 mmHg diastolic blood pressure [DBP]), none were taking medication, had a history of cardiovascular or inflammatory complications, or had a high level of physical training.

### 2.3 Experimental design

On the first visit, the women filled out the following questionnaires: (1) an ad hoc sociodemographic questionnaire about age, educational level, economic situation and global health status, and (2) a self‐report Activity Questionnaire for Adults and Adolescents (AQuAA; Chinapaw et al., 2009), a validated tool (Busschaert et al., 2015), which explores the frequency and effort of common physical or sports leisure activities (Liu et al., 2011). From the AQuAA, the total activities were calculated in metabolic equivalent of tasks (MET) (kcal/kg week). At this appointment, body composition was assessed. A scale‐tallimeter (Tanita WB 380‐H; BioLogica S.L., Barcelona, Spain) was used to measure height and body weight, and waist and hip circumference were also evaluated with a tape measure and impedanciometry (Bodystat 520, SN 510802; BioLogica S.L.) was used. From these measurements, the following parameters were obtained: body mass index (kg/m^2^), waist to hip index (WHI, a.u.), fat mass (% kg), muscle mass (% kg), total body water (L), and basal metabolic rate (kcal/day).

**FIGURE 1:**
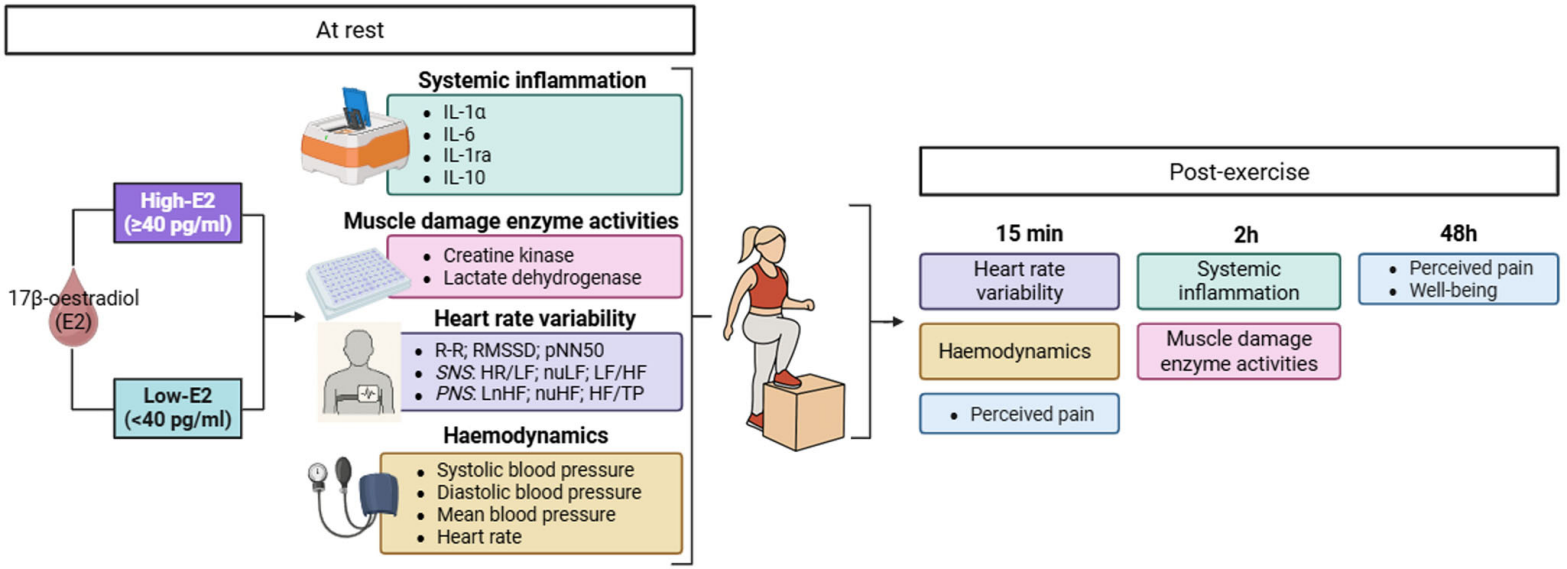
Study design. E2, 17β‐Oestradiol; HF, high frequency power; IL, interleukin; LF, low frequency power; pNN50, percentage of consecutive normal sinus intervals that differ >50 ms; PNS, parasympathetic nervous system; RMSSD, root mean square of successive differences of R–R intervals; SNS, sympathetic nervous system; TP, total power.

On the next visit, the women were instructed to refrain from strenuous exercise the day before. During this appointment, women were asked about the phase of their menstrual cycle, considering menstruation as a sign and a cycle of 28–35 days. Accordingly, women were stratified into follicular phase (days 1–8 and days 25+) and luteal phase (days 9–24). The women were also asked about the use of hormone‐based contraception. Cardiovascular parameters (blood pressure and HRV) were measured at rest and 15 min post‐exercise. Blood samples were taken at rest and 2 h post‐exercise. Pain perception was assessed immediately after exercise and 48 h post‐exercise, together with well‐being perception. Figure 1 shows experimental design.

### 2.4 Exercise protocol

The exercise protocol consisted of repeated step‐up and step‐down movements on a plyometric box (Vevor ES, Madrid, Spain) with a height approximately equal to the distance from the participant's knee to the ground. The stepping frequency was set at 15 cycles per minute and regulated using a metronome, as previously described (Larsen et al., 2007). The exercise was monitored by a staff member with a metronome, ensuring larger duration (3 s) of the down step to guarantee an eccentric component of the exercise. In addition, a health professional and member of the research team attended to the participants throughout the entire exercise protocol. The exercise was performed with both legs until volitional exhaustion, as assessed using the Borg Rating of Perceived Exertion scale (Borg, 1982), and the number of complete repetitions (up and down steps) was annotated.

### 2.5 Measurements

#### 2.5.1 Perceptual pain and well‐being responses questionnaire

##### 2.5.1.1 Pain perception assessment

Soreness was evaluated immediately after exercise completion and 48 h post‐exercise (through a phone call) by a questionnaire as previously described (Tavares et al., 2018). The questionnaire used a Likert scale of 1–5, with 0.5‐point increments (from 1: no pain to 5: severe pain), from 20 different muscle sites (10 from the left and 10 from the right side). Lower‐body pain perception was calculated as sum of the left and right quadriceps, calves, abdominal, hamstring and gluteus muscles (within a range between 10 and 50 points). The perception of the upper‐body pain was calculated as the sum of left and right chest, shoulder, neck, lower and upper back muscles, (within a range between 10 and 50 points). From these evaluations, total pain perception was calculated from the sum of all muscle regions, with a minimum of 20 and a maximum of 100 points. The higher the score, the larger the pain perception.

##### 2.5.1.2 Perceptual well‐being questionnaire

After 48 h of exercise, women completed the subjective well‐being questionnaire, which assesses fatigue, sleep quality, general muscle soreness, stress levels and mood on a 5‐point Likert scale (1 to 5 with 0.5‐point increments). Overall well‐being was then determined by summing the five scores. The higher the score, the greater the feeling of well‐being or lower perception of fatigue (McLean et al., 2010).

#### 2.5.2 Evaluation of cardiovascular parameters

Cardiovascular parameters were evaluated at rest and 15 min post‐exercise. Systolic and diastolic blood pressure (SBP and DBP; mmHg) were measured using an arm cuff and a digital sphygmomanometer (A&D Medical, San Jose, CA, USA). To take these measures, participants were in a seated position with their back supported, feet flat on the floor, and the measured arm supported at heart level. Two consecutive measurements were obtained, separated by 1 min, and the mean value was used for subsequent analyses. Mean arterial pressure (MAP; mmHg) was calculated as DBP + [(SBP + DBP)/3]. HRV was assessed during a 10‐min supine rest period using a Polar H10 chest strap (Polar Electro Ltd, Kempele, Finland), with electrodes positioned similarly to the V4 ECG lead, tilted slightly to the left below the nipple. The Polar H10 was connected to the ELITE HRV smartphone application (https://elitehrv.com/) for data acquisition, providing the following parameters: (1) time‐domain parameters: heart rate (HR, beats/min), R–R interval (time between consecutive R‐waves in a single heartbeat, s), root mean square of successive differences of R–R intervals (RMSSD, ms), and percentage of successive heartbeats with an interval difference of more than 50 ms (pNN50, %), and (2) frequency‐domain parameters, employing fast Fourier transform and the Welch power spectrum density estimation of low‐frequency power (LF, 0.04–0.15 Hz; ms^2^), high‐frequency power (HF, 0.15–0.40 Hz; ms^2^) and total power (TP, ms^2^). In addition, the HR/LF (Tanoue et al., 2022), LF/HF and HF/TP ratios, normalized LF, as LF/(LF + HF), normalized HF, as HF/(LF + HF), and standardized HF, as ln(HF), were calculated. The guidelines of the Task Force of the European Society of Cardiology and the North American Society of Pacing and Electrophysiology were followed (Malik et al., 1996).

#### 2.5.3 Blood sample extraction and processing

Blood samples were taken at rest and 2 h post‐exercise, which is the time point of significant elevation of inflammation biomarkers (Ramiro‐Cortijo et al., 2025). Briefly, under strict hygiene and safety protocols, a 0.5 mL capillary blood sample was obtained using the Tasso+ M20 device (Tasso Inc., Seattle, WA, USA) connected to a lithium–heparin microtainer tube (Vacutainer, BD, Franklin Lakes, NJ, USA). Blood samples were centrifuged at 4°C at 900* g* for 10 min, and the resulting plasma was stored at –80°C until analysis (volume between 250 and 300 µL).

#### 2.5.4 Plasma 17β‐oestradiol levels

Plasma E2 was evaluated by enzyme‐linked immunosorbent assay (ELISA) (Estradiol Parameter Assay, cat. no. KGE014, R&D Systems, Minneapolis, MN, USA), according to manufacturer's instructions. Briefly, after sample deproteinization with trichloroacetic acid, 100 µL of oestradiol primary antibody solution was added, washed, and a 100 µL volume of standard or sample was mixed with 50 µL of oestradiol conjugate, followed by the addition of substrate solution and further incubation. The optical density was evaluated through a microplate reader (Synergy HT Multi‐Mode, BioTek, Winooski, VT, USA), set to 490 nm. The oestradiol standard curve was used in the range of 3000 to 12.3 pg/mL. According to the manufacturer, the intra‐assay precision was coefficient of variation (CV) 5.4%, the inter‐assay precision was CV 7.6%, and the sensitivity ranged from 2.14 to 12.1 pg/mL. The participants were clustered according to plasma E2 levels into low (<40 pg/mL or approx. <150 pmol/L) and high (≥40 pg/mL or approx. ≥150 pmol/L) according to Frederiksen et al. (2020).

#### 2.5.5 Plasma cytokines

Plasma concentrations of interleukins (IL‐1α, IL‐6, IL‐1ra, IL‐10) were simultaneously quantified in 30 µL plasma samples using a customized cartridge with the Ella™ ultrasensitive multiplex immunoassay system (Bio‐Techne Corp., Minneapolis, MN, USA), following the manufacturer's protocol. This system utilizes microfluidic channels and micro‐reactors pre‐coated with cytokine‐specific detection antibodies to automate duplicate measurements. Fluorescence signals (relative fluorescence units, RFUs) were recorded and used to calculate cytokine concentrations (pg/mL) based on standard curves. The lower and upper limits of quantification (LLOQ and ULOQ, respectively) for each cytokine were as follows: IL‐1α (0.49–1880 pg/mL), IL‐6 (0.28–2652 pg/mL), IL‐1ra (7.37–4500 pg/mL) and IL‐10 (0.38–1446 pg/mL). According to the manufacturer, the inter‐assay precision of the cartridge was CV <20%.

#### 2.5.6 Plasma muscular damage biomarkers

Creatine kinase (CK; cat. no. TK41251) and lactate dehydrogenase (LDH; cat. no. SP41214) activities were used as markers of muscular damage and quantified using commercial kits (Cromakit, Granada, Spain), according to the manufacturer's instructions, with a sensitivity of 1 U/L = 0.0001 ΔAbs/min. All reactions were performed at 37°C, and reading reactions were adjusted to zero against distilled water. The absorbance was measured on the above‐mentioned plate reader.

To determine CK activity (detection limit = 0.878–1300 U/L), 5 µL of plasma was mixed with 250 µL of working reactive (with phosphocreatine + ADP) and incubated for 2 min, which, in the presence of CK produces creatine and ATP. Thereafter, the absorbance was read at 340 nm every minute, for 3 min. The higher the absorbance, the larger the CK activity. CK activity (U/L) was calculated as the difference between absorbances and the average of these differences per minute (ΔAbs/min) × 11.3.

To determine LDH activity (detection limit = 2–1500 U/L), 4.2 µL of plasma was mixed with 250 µL of working reaction mix (with pyruvate + NADH + H^+^) and incubated for 1 min, which in the presence of LDH generates l‐lactate and NAD^+^. Then, the absorbance was read at 340 nm every minute for 3 min. The higher the absorbance, the lower the LDH activity. LDH (U/L) was calculated as the difference between absorbances and the average of these differences per minute (ΔAbs/min) × 13.6. Since CK and LDH measurements can be influenced by muscle mass (Ramiro‐Cortijo et al., 2025), these determinations were related to muscle mass.

### 2.6 Statistical analysis

Statistical analysis was performed using R software (ver. 4.5.0, R Foundation for Statistical Computing, Vienna, Austria) within the RStudio interface (ver. 2025.09.2+418) using *rio*, *CompareGroups*, *ggplot2*, *ggpubr*, *dplyr*, *devtools*, *ggcorrplot*, *effectsize*, and *nlme*, packages.

Quantitative data were expressed as median and interquartile range (IQR) [Q1, Q3]. No lost data imputation techniques were used in this study. Differences between groups (high‐E2 vs. low‐E2) were proved by the Mann–Whitney *U*‐test, using a Bonferroni correction to control for multiple comparisons, and Spearman's rho coefficient (ρ) was used to explore correlations between quantitative variables, examining the bivariate relationships between oestradiol with perceptual pain, cardiovascular parameters, cytokines and muscle damage biomarker concentrations. Additionally, changes in pain, cardiovascular parameters, cytokines or muscle damage biomarkers were analysed by two‐way repeated measurements ANOVA, extracting the main effect between subjects (E2 group; *P*_group_), intra‐subject by time (*P*_time_) and their interaction effect (E2 group × time; *P*_int_). The effect size was calculated as Cohen's *d* in its absolute value, considering small (|*d*|∼0.2); moderate (|*d*|∼0.5) and large (|*d*|∼0.8) effect. The 95% confidence interval of *d* was estimated by its original values (95% CI). The effect size for the repeated measurements ANOVA was calculated by eta‐squared (η^2^), considering small (η^2^ ∼0.01); moderate (η^2^ ∼0.06) and large (η^2^ ∼0.14) effect. Statistical significance was assumed for *P*‐values <0.05, and trends were described when *P *< 0.1.

## 3 RESULTS

### 3.1 Cohort characteristics and oestradiol concentrations

All women were single, had monthly incomes below 500€ and reported occasional or weekend consumption of wine and beer. Participants consumed meals 3–4 times daily and did not report intake of supplements. Three women reported using hormone‐based contraception (progestogen). In the low‐E2 group the plasma level of oestradiol was 12.3 [4.20, 22.5] pg/mL (∼45 pmol/L), and in the high‐E2 group 134.0 [62.9, 252.0] pg/mL (∼490 pmol/L; *P *< 0.001). The self‐reported ovarian cycle day of the women in the low‐E2 group was 6.5 [4.0, 8.8], and in the high‐E2 group 17.0 [15.0, 20.0] days (*P* = 0.004). No significant differences were detected in age, body composition parameters or daily physical activities between women in the low‐E2 and high‐E2 groups (Table 1).

**TABLE 1:** Body composition and physical activity according to plasma oestradiol (E2).

	All (*n* = 36)	Low‐E2 (*n* = 18)	High‐E2 (*n* = 18)	*P*
Age (years)	20.0 [19.0, 24.0]	20.0 [19.0, 24.0]	19.5 [19.0, 22.2]	0.540
Weight (kg)	55.7 [51.8, 60.2]	55.5 [52.5, 59.9]	56.0 [49.8, 60.4]	0.924
BMI (kg/m^2^)	21.4 [19.8, 22.5]	21.1 [19.9, 22.3]	21.4 [20.0, 22.9]	0.739
WHI (a.u.)	0.73 [0.70, 0.75]	0.73 [0.70, 0.74]	0.73 [0.69, 0.75]	0.764
Total body water (L)	28.6 [26.2, 29.2]	28.6 [26.1, 29.2]	28.5 [26.5, 29.3]	0.924
Fat mass (%)	27.7 [26.1, 29.7]	27.7 [26.0, 29.7]	27.6 [26.5, 28.6]	0.899
Muscle mass (%)	33.3 [31.3, 34.5]	32.0 [31.4, 35.0]	33.6 [31.4, 33.8]	0.740
BMR (kcal)	1394 [1307, 1468]	1390 [1323, 1467]	1408 [1295, 1486]	0.862
Total activities (kcal/kg week)	30.9 [20.3, 43.2]	33.3 [23.1, 49.3]	30.5 [19.1, 41.7]	0.402

*Note*: Data show the median and interquartile range [Q1, Q3]. The *P*‐values were by Mann–Whitney's *U* test. Abbreviations: BMI, body mass index; BMR, basal metabolic rate; *n*, sample size; WHI, waist to hip index.

### 3.2 Influence of oestradiol on exercise performance and pain perception

The number of repetitions was not significantly different between groups (low‐E2 = 299 [181, 450] rep., high‐E2 = 298 [160, 404] rep.; *P* = 0.837), and also repetitions normalized by muscle mass showed similar results (low‐E2 = 17.2 [10.9, 23.9] rep./kg muscle, high‐E2 = 16.0 [8.88, 22.5] rep./kg muscle; *P* = 0.752). Oestradiol levels did not correlate significantly with repetitions (ρ = 0.10 [−0.24, 0.42]; *P* = 0.558).

Regarding pain perception immediately (15 min) after exercise, women in the low‐E2 group reported more pain in the lower body than those in the high‐E2 group, this being near statistical significance (*P* = 0.052; |*d*| = 0.35; 95% CI −1.00, 0.31). Lower‐body pain perception at 48 h post‐exercise was increased 10.8% in the low‐E2 group and 0.15% in the high‐E2 group. However, no effect of time or interaction (group × time) was detected, the temporal evolution of pain being similar between groups. For total pain perception, no main effect of group was observed. However, the time effect was near significance (*P* = 0.073; η^2 ^= 0.12), suggesting an increase in pain over time, as expected. At 48 h post‐exercise, women in the low‐E2 group had a 11.2% increase in total pain perception, while in the high‐E2 group it was 1.4%. Again, there was no interaction, and the temporal evolution of total pain was similar in both oestrogen groups (Table 2). Oestradiol levels did not correlate significantly with pain scores.

**TABLE 2:** Pain perception according to plasma oestradiol (E2).

	All (*n* = 36)	Low‐E2 (*n* = 18)	High‐E2 (*n* = 18)	*P*_1_	*P*_group_	*P*_time_	*P*_int_
Lower‐body pain after 15 min (a.u.)	29.0 [22.0, 33.2]	30.0 [23.2, 34.8]	27.5 [20.5, 31.1]	0.304	0.052	0.199	0.369
Lower‐body pain after 48 h (a.u.)	30.0 [26.0, 34.0]	31.2 [26.5, 38.0]	28.0 [26.0, 32.4]	0.099			
Total pain after 15 min (a.u.)	39.0 [33.5, 45.6]	40.0 [34.2, 47.0]	38.0 [31.2, 45.4]	0.631	0.188	0.073	0.301
Total pain after 48 h (a.u.)	40.0 [36.0, 50.5]	42.0 [38.0, 54.9]	39.0 [36.0, 48.1]	0.155			

*Note*: Data show the median and interquartile range [Q1, Q3]. The *P*‐values (*P*_1_) were by Mann–Whitney *U*‐test. The main effect between subjects (E2 group; *P*_group_), intra‐subject by time (*P*_time_) and their interaction effect (E2 group × time; *P*_int_) were by 2‐way repeated measurements ANOVA. Abbreviations: a.u., arbitrary units.

Well‐being scores 48 h after exercise were significantly lower in the low‐E2 group (14.0 [14.0, 15.4] a.u) compared to the high‐E2 group (16.5 [15.0, 18.0] a.u.; *P* = 0.019; |*d*| = 0.83; 95% CI 0.15, 1.51). There was also a significant positive correlation between well‐being scores and oestrogen levels measured on the day of exercise (ρ = 0.58 [0.31, 0.76]; *P *< 0.001).

### 3.3 Systemic inflammatory biomarkers

**FIGURE 2:**
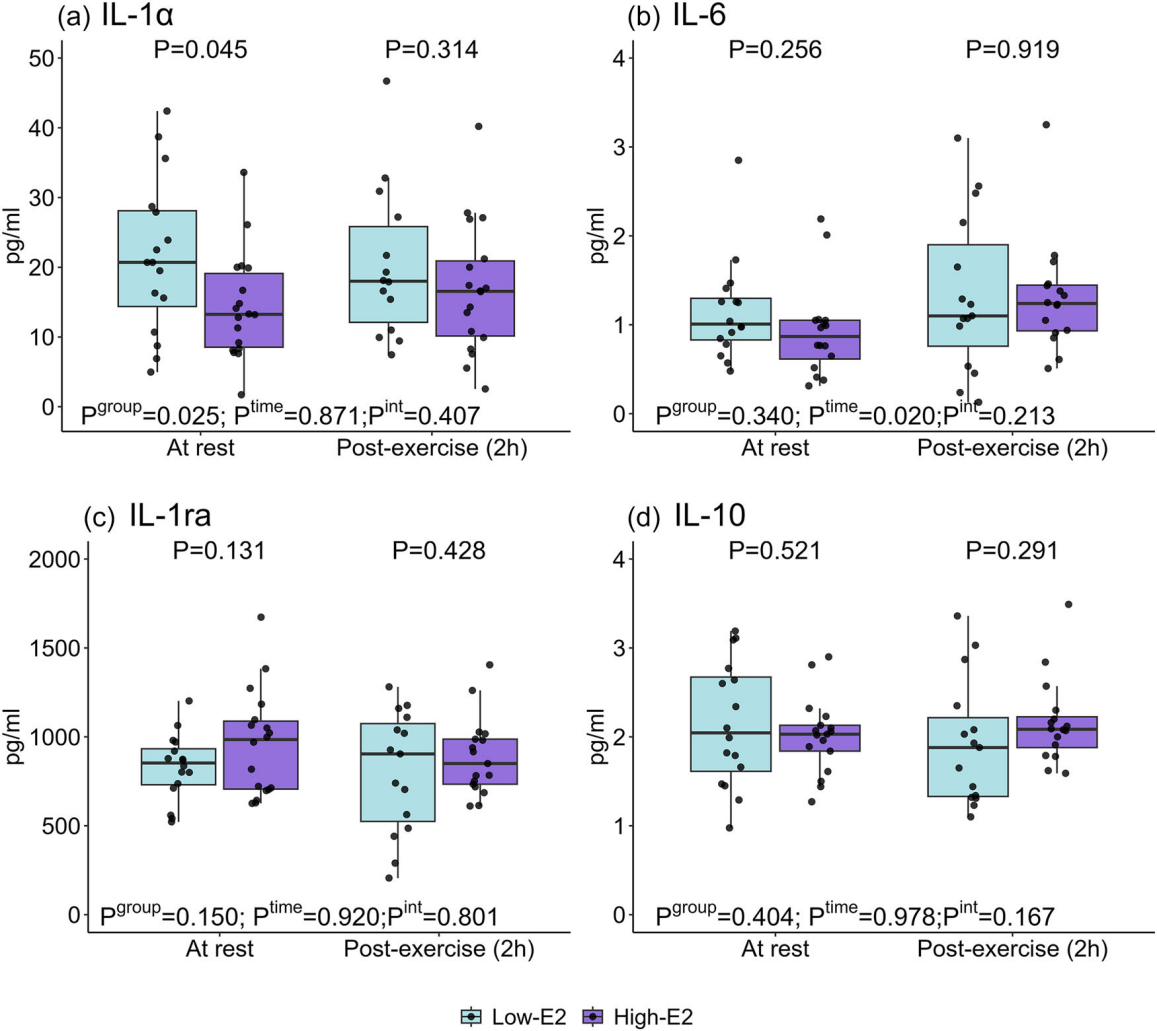
Systemic cytokine biomarkers according to plasma oestradiol (E2). Data show the median and interquartile range [Q1, Q3]. The dots represent each observation. The *P*‐values between groups were obtained by Mann–Whitney *U*‐test adjusted by Bonferroni correction. The main effect between subjects (E2 group; *P*_group_), intra‐subject by time (*P*_time_) and their interaction effect (E2 group × time; *P*_int_) were obtained by 2‐way repeated measurements ANOVA.

At rest, IL‐1α levels were significantly higher in the low‐E2 group compared to the high‐E2 group (*P* = 0.045). There was a significant main effect of E2 group (*P* = 0.025), but no significant differences were observed in time effect or its interaction (Figure 2a). Thus, the exercise stimulus did not modify IL‐1α levels, and the between‐group difference was limited to the resting condition. There was no correlation between oestrogen levels and IL‐1α neither at rest nor 2 h post‐exercise.

IL‐6 levels increased after exercise in both groups, showing a main effect of time (*P*_time_ = 0.020), consistent with a release as myokine in response to exercise (Figure 2b). There were no differences between oestradiol groups and no interaction effect. However, there was a trend to significant and positive correlation with E2 at 2 h post‐exercise (ρ = 0.31 [−0.04, 0.60]; *P* = 0.077).

No significant main or interaction effects were detected for IL‐1ra. Concentrations remained stable from rest to 2 h post‐exercise in both groups (Figure 2c). There was no correlation between oestrogen levels and IL‐1ra either at rest or 2 h post‐exercise. IL‐10 levels did not differ significantly between oestradiol groups and there were no significant changes with time or interaction (Figure 2d). At rest, there was no correlation between oestrogen levels and IL‐10, but there was significant and positive correlation at 2 h post‐exercise (ρ = 0.61 [0.34, 0.79]; *P *< 0.001). In addition, IL‐10 at 2 h post‐exercise negatively correlated with total pain perception at 48 h post‐exercise (ρ = −0.28 [−0.47, −0.06]; *P* = 0.014) and positively correlated with well‐being (ρ = 0.31 [0.09, 0.50]; *P* = 0.006).

### 3.4 Muscle damage biomarkers

**FIGURE 3:**
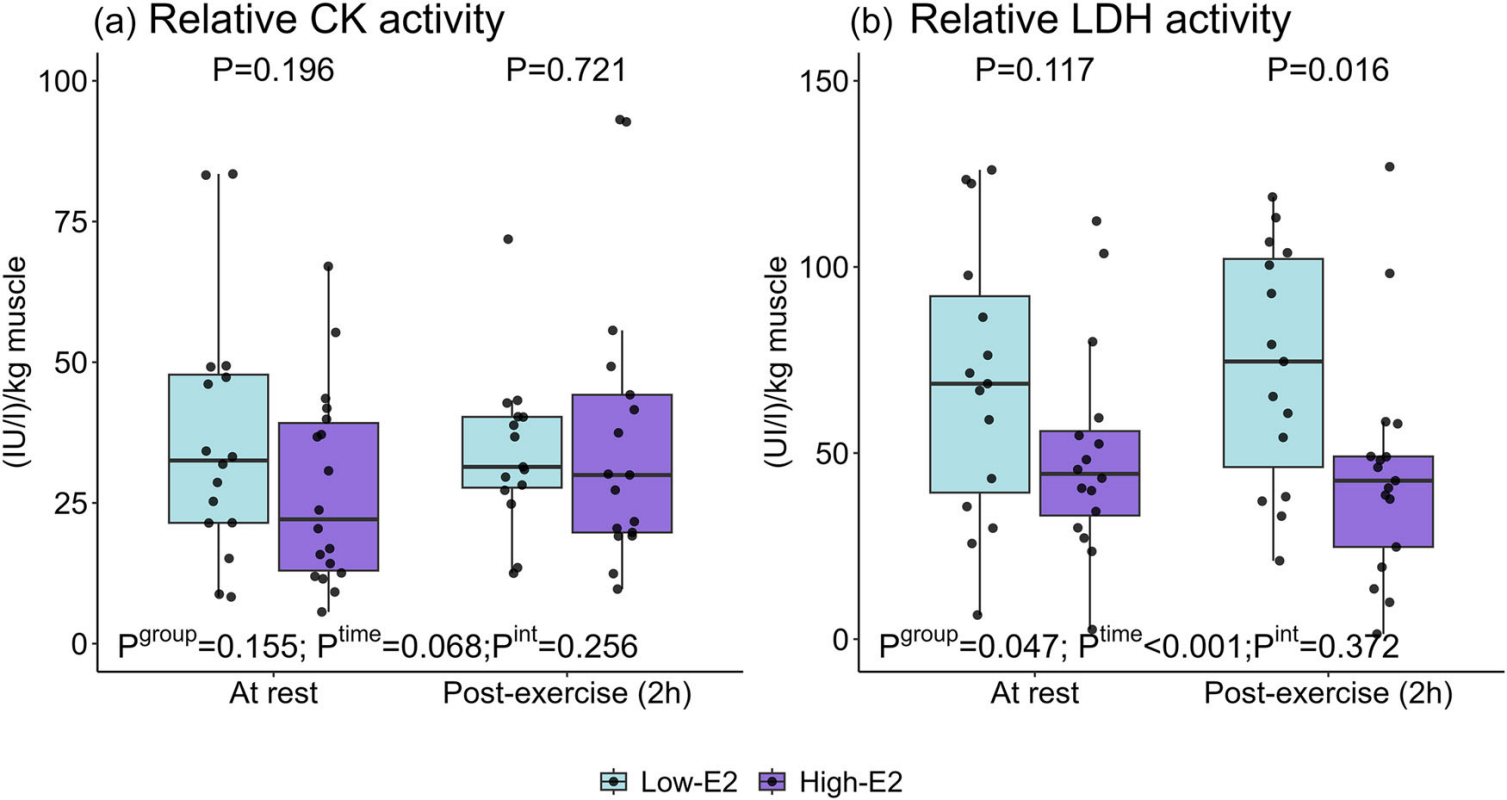
Muscle damage biomarkers according to the plasma oestradiol (E2). Data show the median and interquartile range [Q1, Q3]. The dots represent each observation. The *P*‐values between groups were obtained by Mann–Whitney *U*‐test adjusted by Bonferroni correction. The main effect between subjects (E2 group; *P*_group_), intra‐subject by time (*P*_time_) and their interaction effect (E2 group × time; *P*_int_) were obtained by 2‐way repeated measurements ANOVA.

At rest, CK activity showed no significant difference between oestradiol groups, and no changes were detected after exercise. The repeated‐measures analysis revealed no main effect of group or interaction, but there was a trend toward a main effect of time (*P*_time_ = 0.068), suggesting a slight increase in CK activity following exercise (Figure 3a). There was no correlation between oestrogen levels and CK activity at either rest or 2 h post‐exercise.

At rest, LDH activity showed no significant difference between oestradiol groups (*P* = 0.117). However, 2 h post‐exercise LDH was higher in the low‐E2 group compared with the high‐E2 group after 2 h post‐exercise (*P* = 0.016). In addition, there was a significant main effect of the oestradiol group (*P*_group_ = 0.047) and an effect of time (*P*_time_ < 0.001), indicating that LDH activity increased after exercise in both groups but remained lower in women with higher oestradiol levels. No significant interaction was observed, meaning that the temporal pattern of LDH change was similar across hormonal conditions (Figure 3b). At rest, there was no correlation between oestrogen levels and LDH activity, but there was a significant and negative correlation at 2 h post‐exercise (ρ = −0.41 [−0.66, −0.06]; *P* = 0.019). In addition, the lower‐body pain perception 15 min post‐exercise tended to be significantly and positively correlated with LDH activity at 2 h post‐exercise (ρ = 0.22 [−0.01, 0.42]; *P* = 0.056). LDH activity was also significantly correlated with total pain perception at 48 h (ρ = 0.24 [0.01, 0.44]; *P* = 0.035) and negatively correlated with well‐being (ρ = −0.27 [−0.47, −0.05]; *P* = 0.017).

### 3.5 Cardiovascular parameters and HRV variables

**FIGURE 4:**
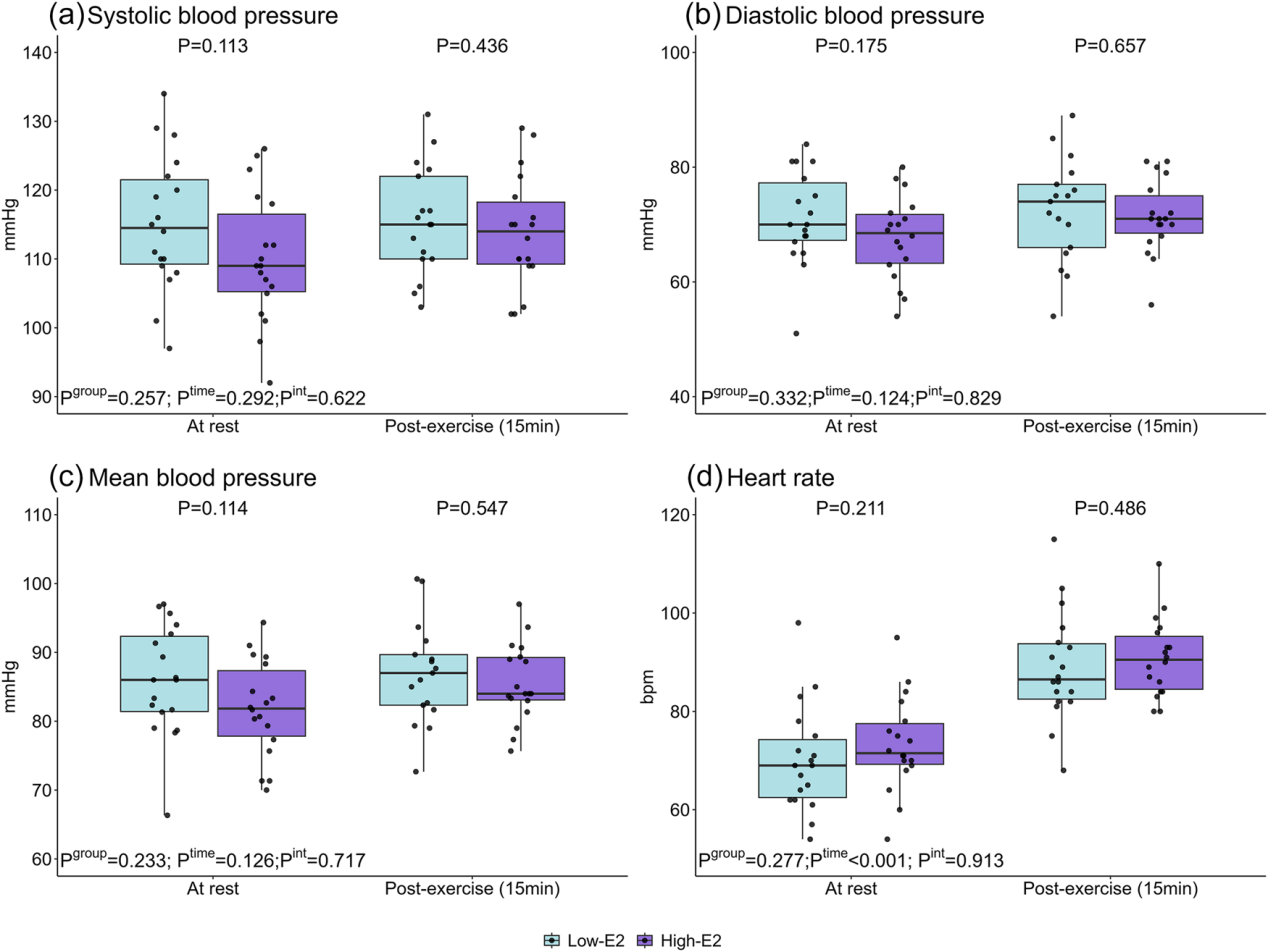
Haemodynamic parameters according to the plasma oestradiol (E2). Data show the median and interquartile range [Q1, Q3]. The dots represent each observation. The *P*‐values between groups were obtained by Mann–Whitney *U*‐test adjusted by Bonferroni correction. The main effect between subjects (E2 group; *P*_group_), intra‐subject by time (*P*_time_) and their interaction effect (E2 group × time; *P*_int_) were obtained by 2‐way repeated measurements ANOVA.

None of the haemodynamic variables were significantly different between groups either at rest or 15 min post‐exercise (Figure 4a–c). There was also no effect of the group, time or their interaction on blood pressure. At rest, there were significant and negative correlations between plasma E2 levels and blood pressure parameters (SBP: ρ = −0.42 [−0.66, −0.11], *P* = 0.010; DBP: ρ = −0.32 [−0.60, 0.01], *P* = 0.058; MAP: ρ = −0.39 [−0.64, −0.08], *P* = 0.017). These correlations were not detected after 15 min post‐exercise.

There was a significant main effect of time on HR (*P*_time_ < 0.001), with no effect of E2 group or interaction, reflecting the expected post‐exercise increase in HR in both hormonal conditions (Figure 4d). Oestrogen levels did not correlate with HR either at rest or 15 min post‐exercise.

**FIGURE 5:**
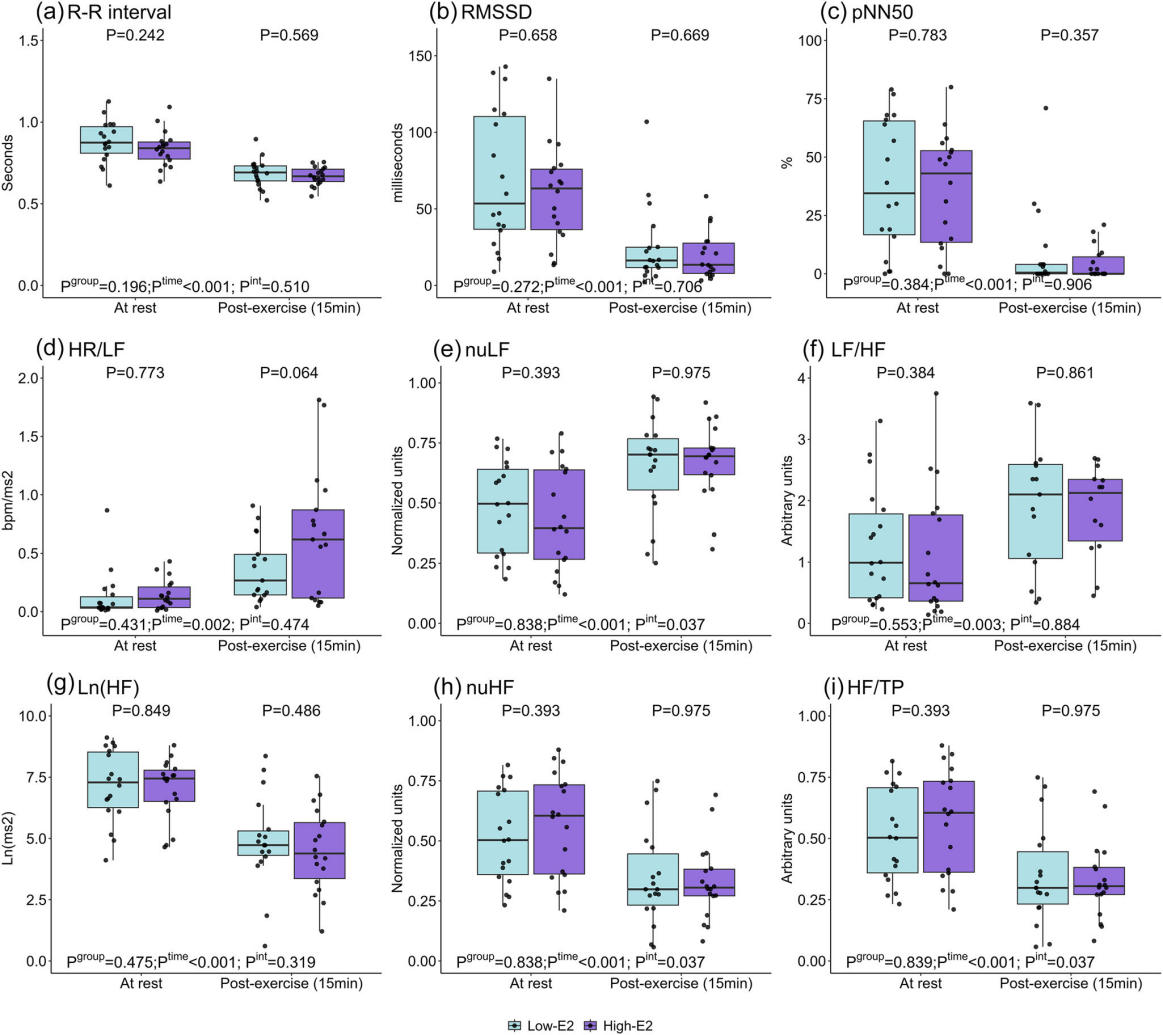
Heart rate variability variables according to plasma oestradiol (E2). Data show the median and interquartile range [Q1, Q3]. The dots represent each observation. The *P*‐values between groups were obtained by Mann–Whitney *U*‐test adjusted by Bonferroni correction. The main effect between subjects (E2 group; *P*_group_), intra‐subject by time (*P*_time_) and their interaction effect (E2 group × time; *P*_int_) were obtained by 2‐way repeated measurements ANOVA. HF, high frequency power; HR, heart rate; LF, low frequency power; pNN5, percentage of successive heartbeats with an interval difference of >50 ms; RMSSD, root mean square of successive differences of R–R intervals; TP, total power.

A significant main effect of time was observed for all HRV parameters, indicating a reduction in PNS modulation and overall HRV 15 min post‐exercise compared with resting values (Figure 5). Specifically, R–R interval, RMSSD, pNN50 and HF significantly decreased post‐exercise, whereas HR/LF and LF/HF ratios increased, consistent with an acute shift toward SNS dominance. No significant main effect of group or interaction was found for any HRV parameter, indicating that the post‐exercise sympathetic nerve activity response was similar between women with low and high E2 groups. Although the high‐E2 group showed a tendency toward higher SNP indices at both time points, these differences did not reach statistical significance. HRV variables were not significantly correlated with E2 levels either at rest or after 15 min post‐exercise.

## 4 DISCUSSION

Studies examining the impact of the ovarian cycle on muscle damage after exercise do not report consistent effects, which has been attributed to different populations (trained or non‐trained), a low number of molecules analysed or difference in assessing the ovarian cycle (reported vs. hormonal evaluation) (Cabre et al., 2024). The focus of the present study is to evaluate EIMD manifestations in a homogeneous population of young women with low level of training, focused on E2, the most prominent and potent oestrogen with muscle repair and membrane protective actions (Enns & Tiidus, 2010; Oosthuyse & Bosch, 2017). We also aimed to address the role of E2 on HRV and capacity to perform exercise.

### 4.1 Influence of oestrogen on exercise performance

The impact of the ovarian cycle has been increasingly recognized as important in sport, particularly regarding muscle strength and performance (Cabre et al., 2024; Hamed‐Hamed et al., 2024; Julian et al., 2017; Kissow et al., 2022). However, most studies have been focused on sport participants, with scarce information on non‐trained women, who do not have muscular adaptations like athletes. The cohort under study was homogeneous in age, health status and was classified as non‐trained based on the METs reported. In this population, the number of repetitions until exertion was taken as exercise performance. We did not find differences between women with high or low E2, and no correlation between repetitions and E2. This is in contrast with the findings in a large cohort of healthy female adult non‐trained volunteers, which evidence that muscle strength increases in follicular phase (Pallavi et al., 2017). In this improvement, the role of E2 is proposed, supported by a systematic review which evidences that muscle strength tends to peak during the ovulatory phase when E2 levels are highest (Kodete et al., 2024). The difference between our study and Pallavi et al. may be related to methodological differences (number of repetitions vs. muscle strength by hand grip test). Therefore, it would be interesting to gain further insight on the influence of E2 using a different research design and testing approach. Although we did not compare the parameters according to the ovarian cycle phases, women in the low‐E2 group were at around the sixth day of the ovarian cycle, corresponding to the early follicular phase, while those in the high‐E2 group were around the 17th day, which could correspond to the ovulatory or luteal phases.

### 4.2 Effect of E2 on pain perception

Ovarian hormone concentrations can also influence pain perception (Romero‐Parra et al., 2021), it being proposed that oestrogens could reduce pain, while progesterone may exert a negative influence, as evidenced in a systematic review (Kodete et al., 2024). Pain after exercise is related to microtrauma of the fibre and the inflammation and swelling, which activates muscle nociceptors (Oosthuyse et al., 2023). Animal studies have demonstrated oestrogen‐mediated attenuation of skeletal muscle inflammation following exercise or injury (Enns & Tiidus, 2010). Moreover, in menopause, ‘musculoskeletal syndrome’ has been coined as a term to relate skeletal muscle pain associated with the loss of oestrogen (Wright et al., 2024). The present study shows a tendency towards less pain in women in the high‐E2 group, immediately after and 48 h post‐exercise. However, no correlation was found between E2 and pain at any time point. It must be considered that nociception is not solely related to oestrogen, but also depends on progesterone and the interaction between these hormones and their receptors (Iacovides et al., 2015). Under our experimental conditions, it was not possible to evaluate progesterone levels, due to limited sample volume, and the evaluation of both hormones is desirable in future studies. Interestingly, we found that the well‐being score at 48 h after exercise was larger in the high‐E2 group, and a positive correlation with E2 was found. The well‐being score evaluates not only general muscle soreness, but also fatigue, sleep quality, stress levels and mood. Conclusively, our results suggest that oestrogen exerts a positive influence on these parameters, while pain on its own may be dependent on the relative amount of E2 and progesterone.

### 4.3 Influence of oestrogen on biomarkers of muscle damage and inflammation

Unaccustomed exercise, particularly involving eccentric contractions, results in microtrauma and muscle fibre membrane disruption. The extent of muscle damage is usually monitored by the release of intercellular enzymes, such as CK and LDH, which are considered good biomarkers of muscle damage under different exercise protocols (Callegari et al., 2017; Jiaming & Rahimi, 2021; Ringleb et al., 2024). Animal studies have demonstrated that oestrogen attenuates muscle membrane disruption and CK release (Enns & Tiidus, 2010). In humans, there are some data supporting a positive influence of oestrogen, evidencing that postmenopausal women on hormone replacement therapy had lower serum CK and LDH activity following eccentric exercise compared to those not taking hormone therapy (Dieli‐Conwright et al., 2009). Besides, a negative correlation between plasma CK and oestrogen levels has been found in women performing downhill running (Oosthuyse et al., 2023). In the women participating in our study, we did not detect an influence of E2 on CK, but higher values of LDH were found post‐exercise in women in the low‐E2 group, together with a negative correlation with oestrogen. Together, these findings indicate that higher oestradiol concentrations may exert a partial protective effect against exercise‐induced muscle membrane disruption and leakage of cytosolic enzymes, which may be related to the effect of oestrogen on membrane stability. The negative correlation between LDH at 2 h and well‐being at 48 h post‐exercise, could support the relationship between muscle fibre damage, leakage of proteins and pain.

Another characteristic of EIMD is the inflammatory response, associated with the presence of cytokines in the circulation (Ringleb et al., 2024). Under resting conditions, a larger fold change in the pro‐inflammatory cytokine IL‐1α was found in the low‐E2 group. However, we did not find an increase in this cytokine during exercise or a correlation between plasma oestradiol and IL‐1α levels. Similarly, we did not find difference in IL‐1ra, which is the receptor antagonist, between groups or a relationship with E2. There is evidence that during menopause lack of oestrogen increases expression of the IL‐1 related family but mostly in relation to bone and osteoporosis (Cheng et al., 2022). The lack of time effect suggests that under the exercise conditions of the present study IL‐1α is not the main cytokine released, and it is not influenced by E2. Instead, IL‐6 increased significantly after exercise in both groups, and a trend towards a positive correlation between E2 and IL‐6 at 2 h post‐exercise was found. Oestrogen exerts protective effects on muscle damage stimulating fibre repair, in part due to proliferation of satellite cells (Enns & Tiidus, 2010), and although IL‐6 is considered a proinflammatory cytokine, it has an important physiological role as a myokine released during exercise to induce immune cell recruitment for tissue repair (Peake et al., 2015). IL‐6 also stimulates subsequent release of anti‐inflammatory cytokines such as IL‐10 (Małkowska & Sawczuk, 2023), thereby contributing to muscle inflammation resolution and recovery (Pedersen, 2011; Pedersen & Fischer, 2007). Since E2 levels were also positively correlated with IL‐10 at 2 h post‐exercise we suggest that elevation of oestrogen may help to recover muscle faster. This would also explain the high well‐being at 48 h post‐exercise and the positive correlation with E2. Therefore, it would be interesting to evaluate the kinetics of these cytokines along the timeline to complete recovery. We found a negative association between IL‐10 and pain perception, but a positive association with well‐being, suggest that under high E2 conditions during the exercise the IL‐6/IL‐10 repair signalling pathway may be influenced by oestrogen levels. Evaluation of the level of these myokines at 48 h could be desirable to confirm the role of E2 in recovery.

### 4.4 Influence of oestrogen on haemodynamic parameters and HRV

The role of oestrogen on blood pressure is well known, particularly through studies in postmenopausal women, which evidence a rise in blood pressure after loss of E2 (Akhter et al., 2023). Our findings at rest are consistent with this evidence, since a negative correlation between E2 and blood pressure levels was found. However, we did not find an influence of E2 on blood pressure after exercise, as previously described in young women during moderate‐intensity leg exercise, which did not differ between cycle phases (Shiozawa et al., 2023).

We also assessed the potential influence of E2 on HRV, which allows monitoring autonomous nervous function, being widely used in sport (Schaffarczyk et al., 2022). After exercise, the analysis of HRV parameters revealed a pronounced SNS predominance, evidenced by the significant main effect of time across all time‐ and frequency‐domain indices, including HR/LF, which has been regarded as a reliable parameter reflecting SNS activation in exercise (Tanoue et al., 2022). Overall, the present results confirm lack of influence of E2 on HRV either at rest or under exercise conditions. Under resting conditions, several authors have reported differences in HRV along the ovarian cycle, being highest near ovulation and lowest near menses, in both non‐trained (Tenan et al., 2014) and trained female athletes (Sims et al., 2021). Several authors have evidenced the importance of progesterone in this effect (Hamidovic et al., 2023), while E2 does not seem to play a clear role (Schmalenberger et al., 2020), also evidenced in the context of menopause, where oestrogen therapy in postmenopausal women had no effect on pre‐ or post‐exercise HRV values (Harvey et al., 2016), which is in accordance with our results. We could not evaluate progesterone due to low sample volume, which would have been desirable.

### 4.5 Limitations and future directions

The present study has some limitations which could be considered in future studies. First, although we focused the study on oestradiol, it would have been desirable to evaluate progesterone, which was not possible due to limited blood volume. It would have been interesting to assess ${\dot{V}}_{O_{2}\max}$ to get information about the influence of oestrogen on exercise capacity, which was not possible due to lack of equipment, although the small deviation in the number of repetitions until exhaustion suggests homogeneity in the population. To demonstrate the influence of E2 on the level of exertion, assessment on the Borg scale score after a set number of repetitions, in addition to its evaluation at exertion, could be more informative. Thirdly, it would be desirable to validate our results in a larger population, including individuals with different diets or ethnic backgrounds. An alternative would be a within‐subject design evaluating participants at various time points of their ovarian cycle, which could provide additional insight into the association between ovarian cycle phase, hormonal levels and EIMD.

### 4.6 Conclusions

In untrained women, oestrogen levels influence EIMD, particularly reducing pain perception. These effects could be related to skeletal muscle membrane protection and activation of IL‐6/IL‐10 myokine signalling. Oestrogen levels reduce resting blood pressure levels but do not influence HRV, and the role of progesterone on these parameters deserves further attention.

## AUTHOR CONTRIBUTIONS

David Ramiro‐Cortijo and Silvia M. Arribas: Conceived and designed research. David Ramiro‐Cortijo, Ricardo Alonso de Celada, Pilar Rodríguez‐Rodríguez, Stefanny Lozano Gutiérrez, Leonardo Betancourt and Santiago Ruvira: Performed experiments. David Ramiro‐Cortijo and Silvia M. Arribas: Analysed data and interpreted results of experiments. David Ramiro‐Cortijo: Prepared figures and drafted manuscript. David Ramiro‐Cortijo and Silvia M. Arribas: Edited and revised manuscript. All authors have read and approved the final version of this manuscript and agree to be accountable for all aspects of the work in ensuring that questions related to the accuracy or integrity of any part of the work are appropriately investigated and resolved. All persons designated as authors qualify for authorship, and all those who qualify for authorship are listed.

## CONFLICT OF INTEREST

The authors declare no conflicts of interest. The results of the study are presented clearly, honestly, and without fabrication, falsification, or inappropriate data manipulation. The results of the present study do not constitute endorsement by the American College of Sports Medicine.

## Data Availability

All data generated or analysed during this study are included in this published article and its supplementary information files. In addition, the original data may be requested from the corresponding author and will be available after evaluation by the research team, in accordance with institutional requirements and in full compliance with data protection and ethical regulations.
